# Safety Endpoints With Vadadustat Versus Darbepoetin Alfa in Patients With Non–Dialysis-Dependent CKD: A Post Hoc Regional Analysis of the PRO_2_TECT Randomized Clinical Trial of ESA-Naïve Patients

**DOI:** 10.1016/j.xkme.2023.100666

**Published:** 2023-05-12

**Authors:** Wolfgang C. Winkelmayer, Susan Arnold, Steven K. Burke, Glenn M. Chertow, Kai-Uwe Eckardt, Alan G. Jardine, Eldrin F. Lewis, Wenli Luo, Kunihiro Matsushita, Peter A. McCullough, Todd Minga, Patrick S. Parfrey

**Affiliations:** 1 Section of Nephrology, Baylor College of Medicine, Houston, TX; 2 Excellentis Clinical Trial Consultants, South Africa; 3 Akebia Therapeutics Inc, Cambridge, MA; 4 Division of Nephrology, Stanford University School of Medicine, Palo Alto, CA; 5 Department of Nephrology and Medical Intensive Care, Charité–Universitätsmedizin Berlin, Berlin, Germany; 6 Department of Cardiovascular and Medical Sciences, University of Glasgow, Glasgow, UK; 7 Department of Epidemiology, Johns Hopkins Bloomberg School of Public Health, Baltimore, MD; 8 Truth for Health Foundation, Tucson, AZ; 9 Division of Nephrology, Memorial University, St John's, Newfoundland, Canada

**Keywords:** Anemia, chronic kidney disease, hypoxia-inducible factor, vadadustat, darbepoetin alfa

## Abstract

**Rationale & Objective:**

Prespecified analyses of the PRO_2_TECT trials comparing the safety of the oral hypoxia-inducible factor prolyl hydroxylase inhibitor vadadustat with darbepoetin alfa in patients with non–dialysis-dependent chronic kidney disease (NDD-CKD) found no difference in major adverse cardiovascular events (MACE; death from any cause or nonfatal myocardial infarction or stroke) among US patients and a higher risk among patients treated with vadadustat outside the United States. We investigated regional differences in MACE in the PRO_2_TECT trial that enrolled 1,751 patients previously untreated with erythropoiesis-stimulating agents.

**Study Design:**

Phase 3, global, open-label, randomized, active-controlled clinical trial.

**Setting and Participants:**

Erythropoiesis-stimulating agent–untreated patients with anemia and NDD-CKD.

**Intervention:**

Eligible patients were randomized 1:1 to receive vadadustat or darbepoetin alfa.

**Outcomes:**

The primary safety end point was time to first MACE. Secondary safety end points included time to first expanded MACE (MACE plus hospitalization for heart failure or thromboembolic event, excluding vascular access thrombosis).

**Results:**

In the non-US/non-Europe region, there was a higher proportion of patients with baseline estimated glomerular filtration rate (eGFR) level of ≤10 mL/min/1.73 m^2^ in the vadadustat group [96 (34.7%)] than in the darbepoetin alfa group [66 (24.0%)]. In this region, there were 21 excess MACEs reported in the vadadustat group [78 events (n=276)] versus the darbepoetin alfa [57 events (n=275)], including 13 excess noncardiovascular deaths, largely from kidney failure. Noncardiovascular deaths were concentrated in Brazil and South Africa, which enrolled higher proportions of patients with an eGFR of ≤10 mL/min/1.73 m^2^ and who may not have had access to dialysis.

**Limitations:**

Different regional treatment patterns of patients with NDD-CKD.

**Conclusions:**

The higher MACE rate in the non-US/non-Europe vadadustat group may have been partly because of imbalances in the baseline eGFR level in countries where dialysis was not uniformly available resulting in many kidney-related deaths.

## Graphical abstract

**Figure:**
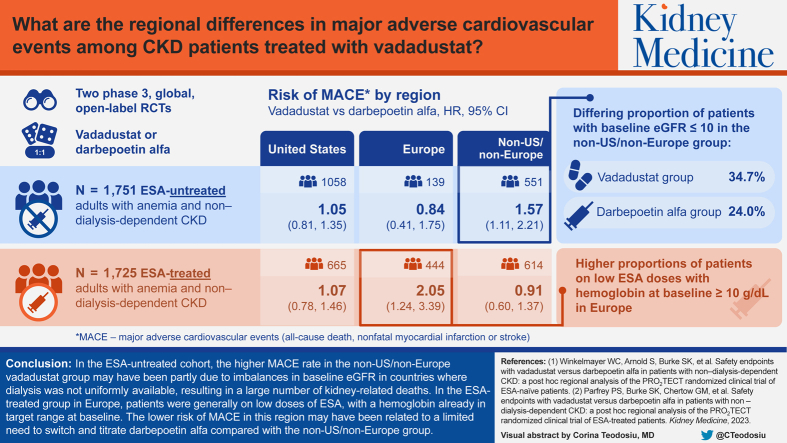

**Plain Language Summary**

Anemia is a common complication of chronic kidney disease (CKD) and associated with reduced quality of life and heightened risk of cardiovascular events. Vadadustat is a novel oral therapy being investigated for treatment of anemia due to CKD. In global phase 3 trials, vadadustat showed noninferiority to injectable erythropoiesis-stimulating agent darbepoetin alfa for time to major adverse cardiovascular events (MACE) in patients with dialysis-dependent CKD but not in those with non–dialysis-dependent CKD. This trial explores regional differences in MACE in erythropoiesis-stimulating agent–naïve patients with non–dialysis-dependent CKD. We found that higher MACE rates in the non-US/non-Europe vadadustat group may have been partly owing to imbalances in the baseline kidney function in countries where dialysis was not uniformly available.

Anemia affects more than half of patients with advanced chronic kidney disease (CKD; stages 4 and 5) and is associated with diminished functional capacity and health-related quality of life.^1^^,^^2^ For patients with non–dialysis-dependent CKD (NDD-CKD), Kidney Disease: Improving Global Outcomes (KDIGO) guideline recommendations suggest that treatment with erythropoiesis-stimulating agents (ESAs) be considered on an individualized basis when the hemoglobin concentration is <10.0 g/dL, with a recommended upper limit for treatment of 11.5 g/dL.^3^ Data from a real-world longitudinal study suggest that patients with NDD-CKD generally receive iron treatment, but initiation of ESA treatment remains limited, and once initiated, the discontinuation rate is high.^4^

In a randomized, placebo-controlled, double-blind clinical trial (Trial to Reduce Cardiovascular Events With Aranesp Therapy [TREAT]), the use of darbepoetin alfa to treat anemia in patients with type 2 diabetes and NDD-CKD did not reduce the risk of death or cardiovascular events and was associated with a near doubling of the risk of ischemic stroke.^5^ TREAT and other trials showing only partial correction of anemia in ESA-treated patients have led to reticence to use ESAs in patients with NDD-CKD.^5^^,^^6^ However, undertreatment of anemia in patients with NDD-CKD may also lead to an increased need for red blood cell transfusions, which may cause allosensitization, thereby reducing a patient’s likelihood of receiving a kidney transplant.^7^

Differing ESA labels across regulatory jurisdictions, changes in reimbursement policies, and variation in guideline recommendations for treatment of anemia associated with CKD have resulted in a high degree of variability in treatment practices.^8^ The Chronic Kidney Disease Outcomes and Practice Patterns Study (CKDopps) reported on country-specific differences in the evaluation and prevalence of anemia, iron deficiency, and related treatment in a study of more than 6,500 patients across 4 countries.^9^ Regional variations in treatment practices have previously been associated with discordant results among geographic regions for major adverse cardiovascular event (MACE) end points within multinational trials investigating interventions for cardiovascular diseases.^10^, ^11^, ^12^, ^13^

We recently completed 4 global phase 3 trials to compare the hematologic efficacy and cardiovascular safety of vadadustat, a hypoxia-inducible factor prolyl hydroxylase inhibitor (HIF-PHI), with that of darbepoetin alfa in patients with NDD-CKD (PRO_2_TECT trials) and dialysis-dependent (DD)-CKD (INNO_2_VATE trials).^14^^,^^15^

In a pooled analysis of patients with NDD-CKD in the 2 PRO_2_TECT trials who were either treated or untreated with ESA, vadadustat was found to be noninferior to darbepoetin alfa for the hematologic efficacy end point but did not meet the noninferiority criteria for end point the cardiovascular safety endpoint of time to first MACE {prespecified noninferiority margin of 1.25 [US Food and Drug Administration (FDA)] and 1.3 [European Medicines Agency]}.^14^ However, a prespecified subgroup analysis suggested that there was no difference in MACE between vadadustat and darbepoetin alfa among patients in the United States [hazard ratio (HR), 1.06; 95% CI, 0.87-1.29], whereas vadadustat was associated with a higher risk of MACE among patients outside the United States (HR, 1.30; 95% CI, 1.05-1.62).^14^ This analysis was performed to investigate regional differences in MACE observed in the PRO_2_TECT trial of ESA-untreated patients.

## Methods

We conducted 2 phase 3, global, open-label (sponsor-blinded), randomized, active-controlled clinical trials, collectively known as the PRO_2_TECT trials, comparing vadadustat with darbepoetin alfa in patients with NDD-CKD who were untreated (ClinicalTrials.gov identifier: NCT02648347) or treated (ClinicalTrials.gov identifier: NCT02680574) with ESA. Trial sites were in North America, Latin America, Europe, Africa, and the Asia-Pacific region. We conducted both trials in compliance with the International Conference on Harmonisation, in accordance with Good Clinical Practice guidelines and FDA regulations, and in line with the principles of the Declaration of Helsinki. We obtained institutional review board approval at each participating center, and all patients provided written informed consent before enrollment. The protocol and primary results for these trials have been reported elsewhere.^14^^,^^15^ A CONSORT diagram showing randomization to either vadadustat or darbepoetin alfa and subsequent trial events is shown for the ESA-untreated PRO_2_TECT trial in Fig S1.

This analysis is limited to patients in 1 of the 2 PRO_2_TECT trials that enrolled patients who were untreated with ESA, that is, not receiving ESA within 8 weeks of enrollment (ClinicalTrials.gov identifier: NCT02648347). Eligible patients in this trial were adults with estimated glomerular filtration rate (eGFR) of ≤60 mL/min/1.73 m^2^, hemoglobin concentration of <10.0 g/dL, serum ferritin of ≥100 ng/mL, and transferrin saturation of ≥20%. The eGFR was calculated using the 2009 Chronic Kidney Disease Epidemiology Collaboration (CKD-EPI) creatinine equation. The eligibility criteria did not include a lower limit for the eGFR; however, patients were to be excluded if they were expected to start dialysis within 6 months from screening. We excluded patients who presented with anemia due to any known cause other than CKD; any red blood cell transfusion within 8 weeks before randomization; active malignancy or a history of active malignancy within 2 years; a recent cardiovascular or thromboembolic event; serum concentrations of alanine aminotransferase, aspartate transaminase, and total bilirubin >2 × upper limit of normal; or uncontrolled hypertension.

We randomized eligible patients 1:1 to receive vadadustat or darbepoetin alfa, stratified by regions (US vs Europe vs non-US/non-Europe; see Table S1 for the list of countries), New York Heart Association (NYHA) heart failure class (0/I vs II/III), and hemoglobin concentration at entry (<9.5 g/dL vs ≥9.5 g/dL). Oral vadadustat was administered at a starting dose of 300 mg once daily, with doses of 150 mg, 450 mg, and 600 mg available for adjustment to a maximum dose of 600 mg daily depending on hemoglobin concentrations (hemoglobin targets: United States, 10-11 g/dL; outside the United States, 10-12 g/dL). Darbepoetin alfa was dosed subcutaneously or intravenously based on the approved local product label for adult patients with NDD-CKD. The calculated adherence rate for a given period was derived from the exposure data as the number of days on dosing in the period as collected on the electronic case report forms divided by the number of days in that period.

The primary safety end point in the prespecified pooled analysis of the 2 PRO_2_TECT trials was time to the first adjudicated MACE, defined as a composite of death from any cause, nonfatal myocardial infarction, or nonfatal stroke. Secondary safety end points included time to first expanded MACE (defined as MACE plus hospitalization for heart failure or thromboembolic event, excluding vascular access thrombosis), cardiovascular (traditional) MACE, cardiovascular mortality, and all-cause mortality.

Region (US vs non-US group) was a factor in the prespecified subgroup analysis for the primary MACE end point in the PRO_2_TECT trials.^14^ The post hoc analyses reported in this trial further investigated the relationship between region and primary and secondary MACE. We conducted analyses on the population of patients who received ≥1 dose of trial drug (the safety population) stratified by regions (US vs Europe vs non-US/non-Europe). Region-based and country-based classifications were completed at the time of trial inception.

We assessed MACE according to the Medical Dictionary for Regulatory Activities (MedDRA) version 23.0. We conducted all analyses using the SAS version 9 (SAS Institute Inc). The primary safety analysis was based on all first events that accrued across the 2 NDD-CKD trials (in ESA-untreated and ESA-treated patients). The sample size regarding the MACE end point was determined based on the number of events needed to show noninferiority based on the 2-sided 95% CI for the HR (vadadustat/darbepoetin alfa). We calculated that 631 events would be required overall (in both trials combined) to have 80% power to establish noninferiority with a margin of 1.25 and >90% power to establish noninferiority with a margin of 1.3, assuming no difference between the treatment groups. A MACE rate of 10% annually was anticipated in both treatment groups based on a comprehensive review of available epidemiology and prospective clinical studies in the field.

An analysis of time to first MACE and expanded MACE was performed using a multivariate Cox regression model, including addition of the covariates of baseline hemoglobin concentration, randomization strata of region (US, Europe, and non-US/non-Europe) and NYHA class (0 or I, II, or III), sex (male or female), age, (≤65 or >65 years), race (White or non-White), pre-existing cardiovascular disease (yes/no), and diabetes mellitus (yes/no). Post hoc subgroup analyses reported in this trial explored the following: (1) cumulative incidence of time to the first MACE stratified by regions (US vs Europe vs non-US/non-Europe), (2) time to first MACE adjusting for the baseline eGFR (in addition to the other covariates listed in the primary multivariate Cox regression model), (3) time to first expanded MACE and all-cause mortality stratified by regions, (4) the primary cause of death stratified by regions, (5) treatment-emergent adverse events (TEAEs) stratified by regions, and (6) MACE rates in patients with a baseline eGFR level of ≤10 and >10 mL/min/1.73 m^2^ stratified by regions. Differences in baseline eGFR statuses in patients in non-US/non-Europe, South Africa, and Brazil are reported as mean ± SD (standard deviation), median, lower/upper quartiles, and range category.

## Results

In total, 1,751 patients were randomized in the PRO_2_TECT trial that enrolled ESA-untreated patients, and 1,748 received at least 1 dose of the trial drug (safety population), which comprised 1,058 patients in the United States, 139 in Europe, and 551 outside the United States and Europe. Treatment adherence was ∼99% across all regions in both trial arms. Baseline demographic, clinical, and laboratory characteristics stratified by regions are summarized in Table 1. On average, patients in the non-US/non-Europe group were considerably younger and had lower eGFR than patients in the United States or Europe. Patient characteristics were well balanced across the randomized groups, except for a lower proportion of patients in Europe with diabetes at baseline in the darbepoetin alfa group (42.6% vs 54.9% in the vadadustat group), and a lower (mean ± SD) eGFR in patients randomized to vadadustat in the non-US/non-Europe group (17.1 ± 11.7 mL/min/1.73 m^2^ vs 19.6 ± 13.1 mL/min/1.73 m^2^ in patients randomized to darbepoetin alfa). Ninety-six (34.7%) patients randomized to vadadustat versus 66 (24.0%) patients randomized to darbepoetin alfa in the non-US/non-Europe group had a baseline eGFR of ≤10 mL/min/1.73 m^2^.

**Table 1:** Selected Baseline Demographics, Clinical, and Laboratory Characteristics in ESA-Untreated Patients With NDD-CKD (Safety Population)

Characteristic	Overall		US		Europe		Non-US/non-Europe	
	VADA (n=878)	DA (n=870)	VADA (n=531)	DA (n=527)	VADA (n=71)	DA (n=68)	VADA (n=276)	DA (n=275)
Age, y, mean ± SD	65.2 ± 14.3	64.9 ± 13.7	67.3 ± 13.2	67.4 ± 12.6	70.6 ± 12.8	69.8 ± 11.9	59.6 ± 15.0	58.9 ± 14.2
Female, sex, n (%)	474 (54.0)	506 (58.2)	278 (52.4)	310 (58.8)	35 (49.3)	37 (54.4)	161 (58.3)	159 (57.8)
Race, n (%)^a^								
White	546 (62.2)	570 (65.5)	328 (61.8)	342 (64.9)	66 (93.0)	66 (97.1)	152 (55.1)	162 (58.9)
Black	188 (21.4)	171 (19.7)	157 (29.6)	148 (28.1)	3 (4.2)	1 (1.5)	28 (10.1)	22 (8.0)
Asian	48 (5.5)	37 (4.3)	29 (5.5)	19 (3.6)	2 (2.8)	0 (0.0)	17 (6.2)	18 (6.5)
Native American or Alaska Native	22 (2.5)	23 (2.6)	2 (0.4)	1 (0.2)	0 (0.0)	0 (0.0)	20 (7.2)	22 (8.0)
Other^b^	74 (8.4)	69 (7.9)	15 (2.8)	17 (3.2)	0 (0.0)	1 (1.5)	59 (21.4)	51 (18.5)
eGFR, mL/min/1.73 m^2^								
Mean ± SD	21.2 ± 12.0	21.9 ± 12.6	23.0 ± 11.8	22.6 ± 12.3	22.8 ± 10.5	25.0 ± 12.0	17.1 ± 11.7	19.6 ± 13.1
Median	18	19	21	19	20	24	14	16
Baseline eGFR, mL/min/1.73 m^2^, n (%)								
≤5	24 (2.7)	21 (2.4)	4 (0.8)	6 (1.1)	0 (0.0)	0 (0.0)	20 (7.2)	15 (5.5)
>5 to ≤10	134 (15.3)	117 (13.4)	51 (9.6)	61 (11.6)	7 (9.9)	5 (7.4)	76 (27.5)	51 (18.5)
>10 to ≤15	185 (21.0)	184 (21.1)	109 (20.5)	102 (19.4)	12 (16.9)	11 (16.2)	64 (23.2)	71 (25.8)
>15 to ≤20	152 (17.3)	171 (19.6)	100 (18.8)	116 (22.0)	17 (23.9)	9 (13.2)	35 (12.7)	46 (16.7)
>20	383 (43.6)	377 (43.3)	267 (50.3)	242 (45.9)	35 (49.3)	43 (63.2)	81 (29.3)	92 (33.5)
Disease history, n (%)								
Diabetes mellitus	581 (66.2)	597 (68.6)	388 (73.1)	393 (74.6)	39 (54.9)	29 (42.6)	154 (55.8)	175 (63.6)
Cardiovascular disease	406 (46.2)	411 (47.2)	261 (49.2)	266 (50.5)	39 (54.9)	38 (55.9)	106 (38.4)	107 (38.9)
Hemoglobin, g/dL, mean ± SD	9.1 ± 0.8	9.1 ± 0.8	9.1 ± 0.7	9.1 ± 0.7	9.4 ± 0.6	9.5 ± 0.6	9.0 ± 0.9	9.0 ± 0.9
Blood pressure, mm Hg								
Systolic	139.1 (18.5)	139.1 (17.7)	139.8 (18.3)	139.7 (17.9)	135.4 (16.5)	135.8 (13.3)	138.8 (19.4)	138.7 (18.4)
Diastolic	73.9 (12.0)	73.3 (12.6)	71.8 (12.0)	70.4 (12.3)	75.4 (10.8)	75.7 (10.0)	77.6 (11.6)	78.3 (12.0)
Supplemental iron use, n (%)								
Not receiving any iron	482 (54.9)	467 (53.7)	267 (50.3)	263 (49.9)	54(76.1)	46 (67.6)	161 (58.3)	158 (57.5)
Receiving intravenous iron only	22 (2.5)	20 (2.3)	16 (3.0)	12 (2.3)	0 (0.0)	1 (1.5)	6 (2.2)	7 (2.5)

Abbreviations: NDD-CKD ESA, non–dialysis-dependent chronic kidney disease erythropoiesis-stimulating agent; VADA, vadadustat; DA, darbepoetin alfa; SD, standard deviation; eGFR, estimated glomerular filtration rate.

a Race and ethnic group were reported by the patient.

b Native Hawaiian or other Pacific islander, other, multiple, or not reported.

**Figure 1:**
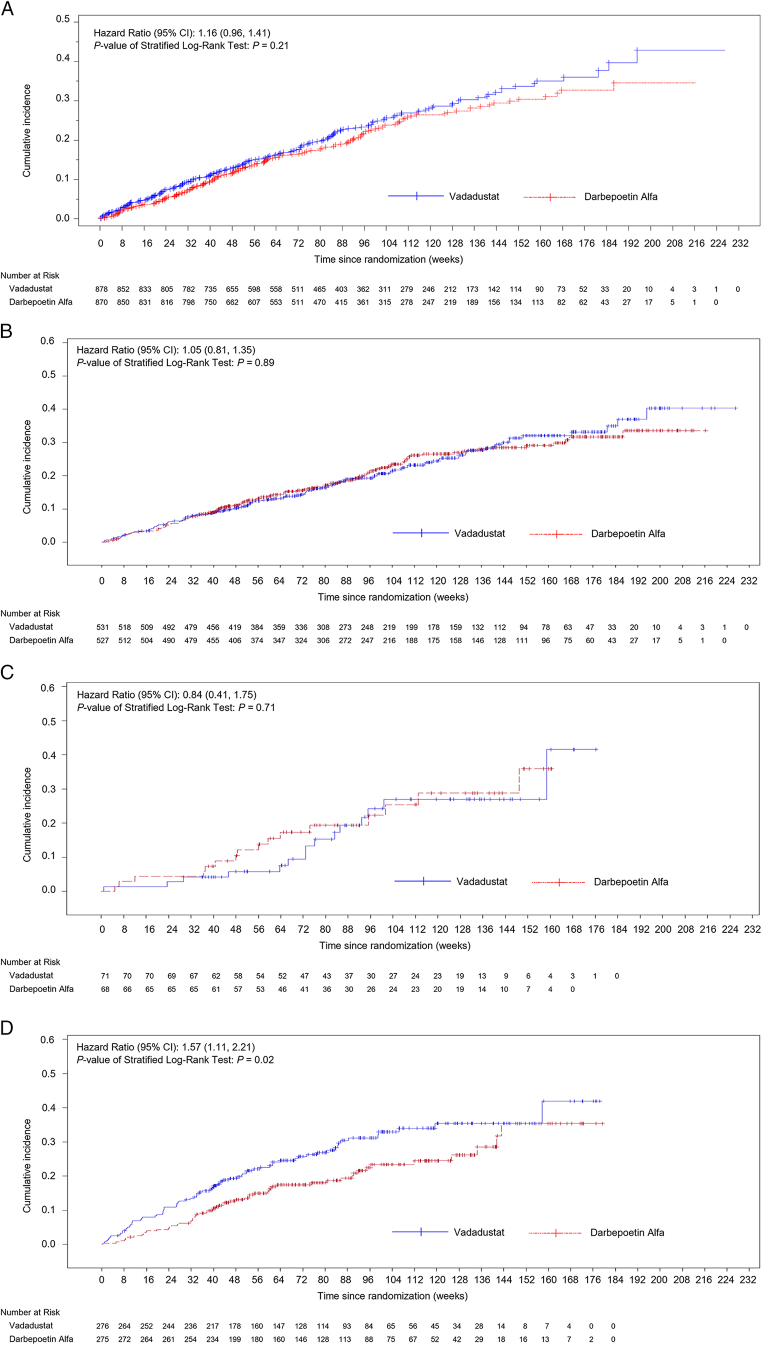
MACE in ESA-untreated patients by regions: (A) overall regions; (B) United States; (C) Europe; (D) non-US/non-Europe. ESA, erythropoiesis-stimulating agent; MACE, major adverse cardiovascular event.

The HR for MACE in the overall population for vadadustat versus darbepoetin alfa was 1.16; 95% CI, 0.96-1.41 (Fig 1; Table 2). When adjusting for the baseline eGFR status, the HR for MACE in the overall population for vadadustat versus darbepoetin alfa was 1.16; 95% CI, 0.95-1.41. When analyzed by regions, the event rates for MACE within the first 2 years of the trial were higher in the non-US/non-Europe group (Table 2) than those in the US or Europe groups. Similar trends in HR were found for the expanded MACE and all-cause mortality across the trial regions (Table S2).

**Table 2:** Cumulative Incidence First MACE in ESA-Untreated Patients with NDD-CKD by Regions (Safety Population)

Statistics	Overall		US		Europe		Non-US/non-Europe	
	VADA (n=878)	DA (n=870)	VADA (n=531)	DA (n=527)	VADA (n=71)	DA (n=68)	VADA (n=276)	DA (n=275)
Cumulative incidence (95% CI)								
52 wk	0.14 (0.12-0.16)	0.13 (0.11-0.15)	0.11 (0.09-0.14)	0.13 (0.10-0.16)	0.06 (0.02-0.15)	0.12 (0.06-0.23)	0.21 (0.17-0.27)	0.14 (0.10-0.18)
104 wk	0.25 (0.22-0.29)	0.24 (0.21-0.27)	0.21 (0.18-0.26)	0.24 (0.20-0.28)	0.27 (0.17-0.42)	0.25 (0.15-0.40)	0.33 (0.27-0.40)	0.23 (0.18-0.30)
156 wk	0.34 (0.29-0.38)	0.30 (0.26-0.35)	0.32 (0.27-0.38)	0.29 (0.25-0.34)	0.27 (0.17-0.42)	0.36 (0.21-0.57)	0.35 (0.29-0.43)	0.35 (0.25-0.48)
208 wk	0.43 (0.35-0.52)	0.35 (0.29-0.41)	0.40 (0.32-0.50)	0.34 (0.28-0.41)	NA^a^	NA^a^	NA^a^	NA^a^
Hazard ratio (vadadustat/darbepoetin alfa) (95% CI)	1.16 (0.96-1.41)		1.05 (0.81-1.35)		0.84 (0.41-1.75)		1.57 (1.11-2.21)	

Abbreviations: MACE, major adverse cardiovascular event; NDD-CKD ESA, non–dialysis-dependent chronic kidney disease erythropoiesis-stimulating agent; VADA, vadadustat; DA, darbepoetin alfa; CI, confidence interval; NA, not available.

a CI estimates are not available for Europe and non-US/non-Europe given that no patients reached this time point in these regions.

Outside the United States and Europe, 551 patients, of whom 135 had a primary MACE, were randomized across 11 countries. The net difference in MACE in the non-US/non-Europe group was 21 excess events reported in the vadadustat group [vadadustat, 78 events (n=276)] versus darbepoetin alfa group [57 events (n=275)]. Death events accounted for 27% of the MACEs (n=75/276) in the vadadustat group and 20% of the MACEs (n=55/275) in the darbepoetin alfa group. Noncardiovascular deaths accounted for 43 and 30 events, respectively (Table 3). Most of these noncardiovascular deaths were attributed to kidney failure (n=25/43 in the vadadustat group; n=20/30 in the darbepoetin alfa group).

**Table 3:** Summary of Primary Cause of Death in ESA-Untreated Patients with NDD-CKD by Regions (Safety Population)

Primary Cause of Death	US, n (%)/(per 100 PY)		Europe, n (%)/(per 100 PY)		Non-US/non-Europe, n (%)/(per 100 PY)	
	VADA (n=531/PY = 1014.4)	DA (n=527/PY = 1009.0)	VADA (n=71/PY = 126.9)	DA (n=68/PY = 122.5)	VADA (n=276/PY = 391.9)	DA (n=275/PY = 423.0)
CV death	40 (7.5)/(3.9)	38 (7.2)/(3.8)	7 (9.9)/(5.5)	8 (11.8)/(6.5)	24 (8.7)/(6.1)	19 (6.9)/(4.5)
Non-CV death	42 (7.9)/(4.1)	47 (8.9)/(4.7)	4 (5.6)/(3.2)	3 (4.4)/(2.4)	43 (15.6)/(11.0)	30 (10.9)/(7.1)
Renal	13 (2.4)/(1.3)	15 (2.8)/(1.5)	2 (2.8)/(1.6)	0 (0)/(0)	25 (9.1)/(6.4)	20 (7.3)/(4.7)
Infection	10 (1.9)/(1.0)	8 (1.5)/(0.8)	1 (1.4)/(0.8)	2 (2.9)/(1.6)	10 (3.6)/(2.6)	6 (2.2)/(1.4)
GI	3 (0.6)/(0.3)	6 (1.1)/(0.6)	1 (1.4)/(0.8)	0 (0)/(0)	3 (1.1)/(0.8)	1 (0.4)/(0.2)
Other non-CV	4 (0.8)/(0.4)	5 (0.9)/(0.5)	0 (0)/(0)	0 (0)/(0)	2 (0.7)/(0.5)	0 (0)/(0)
Malignancy	4 (0.8)/(0.4)	7 (1.3)/(0.7)	0 (0)/(0)	1 (1.5)/(0.8)	1 (0.4)/(0.3)	2 (0.7)/(0.5)
Pulmonary	4 (0.8)/(0.4)	4 (0.8)/(0.4)	0 (0)/(0)	0 (0)/(0)	1 (0.4)/(0.3)	0 (0)/(0)
Accidental	4 (0.8)/(0.4)	2 (0.4)/(0.2)	0 (0)/(0)	0 (0)/(0)	1 (0.4)/(0.3)	1 (0.4)/(0.2)
Unknown	10 (1.9)/(1.0)	16 (3.0)/(1.6)	2 (2.8)/(1.6)	1 (1.5)/(0.8)	8 (2.9)/(2.0)	6 (2.2)/(1.4)

Abbreviations: NDD-CKD ESA, non–dialysis-dependent chronic kidney disease erythropoiesis-stimulating agent; PY, patient-year; VADA, vadadustat; DA, darbepoetin α; CV, cardiovascular; GI, gastrointestinal.

The top 5 enrolling countries in the non-US/non-Europe group were Ukraine (131 patients), Brazil (120 patients), South Africa (103 patients), Mexico (91 patients), and Argentina (51 patients), contributing 90% of the non-US/non-European trial population. Sixty-seven (53.6%) of the 125 deaths and 19 excess deaths in the non-US/non-Europe group reported for the vadadustat group compared with those in the darbepoetin alfa group were observed in 2 of these countries: Brazil and South Africa.

A high proportion of patients in Brazil and South Africa had an eGFR of ≤10 mL/min/1.73 m^2^ at randomization, and patients in these countries were more severely anemic. Despite randomization, baseline kidney function was not well balanced across treatment groups in these 2 countries; the mean eGFR was lower (Brazil, 18.1 vs 23.1 mL/min/1.73 m^2^; South Africa, 14.9 vs 17.5 mL/min/1.73 m^2^), and the proportion of patients with baseline eGFR level of ≤10 mL/min/1.73 m^2^, a level at which dialysis is generally indicated, was higher in patients randomized to the vadadustat group (Brazil, 29.0% vs 12.1%; South Africa, 53.9% vs 35.3%) (Table 4). Furthermore, of the 103 patients treated in South Africa, 46 (44.7%) were enrolled with a baseline eGFR level of ≤10 mL/min/1.73 m^2^, and only 6 (13.0%) of those patients were initiated on maintenance dialysis during the trial. When patients were stratified by the baseline eGFR level, we found a significant interaction effect between the eGFR and treatment group (*P* < 0.001), indicating that a lower baseline eGFR level may partially contribute to the increased risk of MACE in patients treated with vadadustat outside the United States and Europe. The HR in the non-US/non-Europe region in the eGFR of ≤10 mL/min/1.73 m^2^ was 1.79; 95% CI, 1.01-3.20. However, the risk of MACE remained elevated (although attenuated) in patients with eGFR of >10 mL/min/1.73 m^2^ from the non-US/non-Europe region, with an HR of 1.46; 95% CI, 0.93-2.30 (Table S3).

**Table 4:** Baseline eGFR in ESA-Untreated Patients in Non-US/Non-Europe, South Africa, and Brazil (Safety Population)

eGFR, mL/min/1.73 m^2^	All Non-US/non-Europe		Brazil		South Africa	
	VADA (n=276)	DA α (n=275)	VADA (n=62)	DA (n=58)	VADA (n=52)	DA (n=51)
Mean ± SD	17.1 ± 11.7	19.6 ± 13.1	18.1 ± 10.3	23.1 ± 12.7	14.9 ± 15.0	17.5 ± 13.3
Median (IQR)	14.0 (9.0-22.0)	16.0 (11.0, 26.0)	14.0 (10.0-28.0)	19.0 (12.0-30.0)	10.0 (8.0-18.5)	13.0 (9.0-+24.0)
Range	2.0-98.0	3.0-76.0	5.0-45.0	7.0-55.0	2.0-98.0	3.0-71.0
Category, n (%)						
0-5	20 (7.2)	15 (5.5)	2 (3.2)	0 (0.0)	8 (15.4)	8 (15.7)
>5-10	76 (27.5)	51 (18.5)	16 (25.8)	7 (12.1)	20 (38.5)	10 (19.6)
>10-15	64 (23.2)	71 (25.8)	15 (24.2)	14 (24.1)	7 (13.5)	10 (19.6)
>15-20	35 (12.7)	46 (16.7)	9 (14.5)	10 (17.2)	5 (9.6)	7 (13.7)
>20	81 (29.3)	92 (33.5)	20 (32.3)	27 (46.6)	12 (23.1)	16 (31.4)

Abbreviations: eGFR, estimated glomerular filtration rate; ESA, erythropoiesis-stimulating agent; VADA, vadadustat; DA, darbepoetin alfa; SD, standard deviation; IQR, interquartile range.

Total adverse events for patients with NDD-CKD who were untreated with ESA have been reported previously.^14^ The adverse event profiles for vadadustat and darbepoetin alfa were generally comparable across regions (Table 5).

**Table 5:** TEAE by Regions (Safety Population)

	US				Europe				Non-US/Non-Europe			
	VADA (n=861; 1,688.6 PY)		DA (n=862; 1,709.2 PY)		VADA (n=295; 522.6 PY)		DA (n=288; 538.1 PY)		VADA (n=583; 902.2 PY)		DA (n=582; 927.0 PY)	
	n (%)	Events (events per 100 PY)	n (%)	Events (events per 100 PY)	n (%)	Events (events per 100 PY)	n (%)	Events (events per 100 PY)	n (%)	Events (events per 100 PY)	n (%)	Events (events per 100 PY)
Any TEAEs	789 (91.6)	8,097 (479.5)	798 (92.6)	8,233 (481.7)	249 (84.4)	1,577 (301.8)	238 (82.6)	1,447 (268.9)	527 (90.4)	3,265 (361.9)	517 (88.8)	3,413 (368.2)
Any drug-related TEAE	86 (10.0)	133 (7.9)	52 (6.0)	73 (4.3)	29 (9.8)	42 (8.0)	9 (3.1)	9 (1.7)	80 (13.7)	126 (14.0)	40 (6.9)	47 (5.1)
Any severe TEAE	466 (54.1)	1,529 (90.5)	462 (53.6)	1,330 (77.8)	111 (37.6)	200 (38.3)	89 (30.9)	164 (30.5)	238 (40.8)	493 (54.6)	232 (39.9)	497 (53.6)
Any SAE	584 (67.8)	2,124 (125.8)	586 (68.0)	2,079 (121.6)	175 (58.6)	408 (78.1)	145 (50.3)	349 (64.9)	320 (54.9)	731 (81.0)	318 (54.6)	740 (79.8)
Any drug-related SAE	14 (1.6)	16 (0.9)	16 (1.9)	18 (1.1)	3 (1.0)	3 (0.6)	2 (0.7)	2 (0.4)	19 (3.3)	21 (2.3)	6 (1.0)	6 (0.6)
Any TEAE leading to trial drug discontinuation	93 (10.8)	121 (7.2)	79 (9.2)	105 (6.1)	29 (9.8)	43 (8.2)	14 (4.9)	19 (3.5)	41 (7.0)	43 (4.8)	11 (1.9)	12 (1.3)
Any drug-related TEAE leading to trial drug discontinuation	10 (1.2)	11 (0.7)	3 (0.3)	4 (0.2)	5 (1.7)	5 (1.0)	1 (0.3)	1 (0.2)	14 (2.4)	15 (1.7)	2 (0.3)	2 (0.2)
Any TEAE leading to death	149 (17.3)	149 (8.8)	166 (19.3)	166 (9.7)	51 (17.3)	51 (9.8)	36 (12.5)	36 (6.7)	112 (19.2)	112 (12.4)	100 (17.2)	100 (10.8)

Abbreviations: TEAE, treatment-emergent adverse event; VADA, vadadustat; DA, darbepoetin alfa; PY, patient-year; SAE, serious adverse event.

## Discussion

In the registrational trials of vadadustat, the orally administered HIF-PHI vadadustat met its prespecified safety end point for noninferiority in MACE in patients receiving dialysis in the INNO_2_VATE trials^15^ but did not meet the same prespecified safety end point in patients not receiving dialysis in the PRO_2_TECT trials.^14^ Approximately half of the patients with NDD-CKD were treated with ESA and approximately half were untreated with ESA before entering the respective trials.^14^ The purpose of this analysis was to investigate the regional differences in MACE in the PRO_2_TECT trial of ESA-untreated patients. For analyses that investigate regional differences in MACE in the PRO_2_TECT trial of ESA-treated patients, please see our companion article.^16^

In the analyses presented earlier, we showed detailed data stratified by regions in the trial of patients who were ESA untreated before trial entry. We found a higher rate of MACE among patients outside the United States and Europe randomized to vadadustat. After observing these findings, we conducted a more in-depth analysis to understand whether there were imbalances in randomization at a regional or country level that might have contributed to these findings. We noted higher rates of noncardiovascular death, predominantly attributable to kidney failure, which seemed to be concentrated among patients in Brazil and South Africa. Many of the patients enrolled in Brazil and South Africa began the trial with severely reduced eGFR. Speculatively, these patients did not have access to ESAs in advance of the trial or to lifesaving dialysis before or after, and getting access to anemia therapy might have motivated their trial participation. Although differences in practice would likely affect patients irrespective of the group to which they were randomized, the mean eGFR was lower and the proportion of patients with severely impaired kidney function (eGFR <10 mL/min/1.73 m^2^) was higher in patients randomized to vadadustat in regions outside the United States and Europe (including Brazil and South Africa). However, this only partially explains the higher rate of MACE in these regions. A subgroup analysis revealed that although there was a significant interaction between the baseline eGFR level and treatment group, a higher rate of MACE remained in patients treated with vadadustat who showed an eGFR of >10 mL/min/1.73 m^2^ and who were located outside the United States and Europe.

We found no evidence that randomization was violated. The inclusion criterion for age was ≥18 years and there was no lower limit on the inclusion criterion for the eGFR, but randomization was not stratified by age or eGFR. Given these factors and the modest number of participants randomized in the non-US/non-Europe group, there is a sizable likelihood of imbalanced randomization, which could have contributed to the observed findings, particularly in regions where dialysis was not uniformly available to patients who progressed to kidney failure. A potential limitation of the trial is that the protocol advised the use of local darbepoetin alfa labeling for dose adjustments, and because these differ slightly across regions, this had the potential to result in variability in darbepoetin alfa dosing among jurisdictions. The trial may have also been limited by the relatively low number of European patients in comparison with those of other regions.

In retrospect, we should have carefully considered the implications of randomizing patients with stage 5 NDD-CKD in regions in which dialysis was not uniformly available and where patients were enrolled with exceptionally low eGFR, who, in most other participating countries, might have already received dialysis or kidney transplantation. We would advise other investigators conducting research in advanced NDD-CKD to avoid this situation in the future. Although research should in general be globally inclusive^17^ and conducting trials in less affluent countries can provide patients with access to desperately needed medical care and attention (and can help investigators and sponsors meet enrollment and timeline targets), downstream consequences of doing so for patients, and for the research in which they participate, must be very carefully considered.

In summary, regional differences in time to MACE were observed in patients with NDD-CKD who were not previously treated with ESAs and were randomized to receive vadadustat or darbepoetin alfa as part of the PRO_2_TECT program. The higher event rate in patients randomized to vadadustat in the non-US/non-Europe group may have been related to imbalances in randomization and design and methodological issues. Across these regions (such as Brazil and South Africa), dialysis was not available for many enrolled participants, rendering them at risk for death from uremia, and patients randomized to vadadustat showed poorer kidney function at baseline than patients randomized to darbepoetin alfa. These findings should inform the context within which the safety of vadadustat and the design of future clinical trials evaluating a range of interventions in patients with advanced NDD-CKD are assessed.
