# Supplementary material for: Safety Endpoints With Vadadustat Versus Darbepoetin Alfa in Patients With Non–Dialysis-Dependent CKD: A Post Hoc Regional Analysis of the PRO2TECT Randomized Clinical Trial of ESA-Naïve Patients
Source: Kidney Med. 2023 May 12;5(7):100666. doi: 10.1016/j.xkme.2023.100666 (PMC10329162; doi:10.1016/j.xkme.2023.100666)
Supplement: Supplementary File (PDF) — Figure S1; Table S1-S3. [file mmc1.pdf]

Fig S1. CONSORT flow chart for the ESA-untreated PRO$_2$TECT NDD-CKD trial.

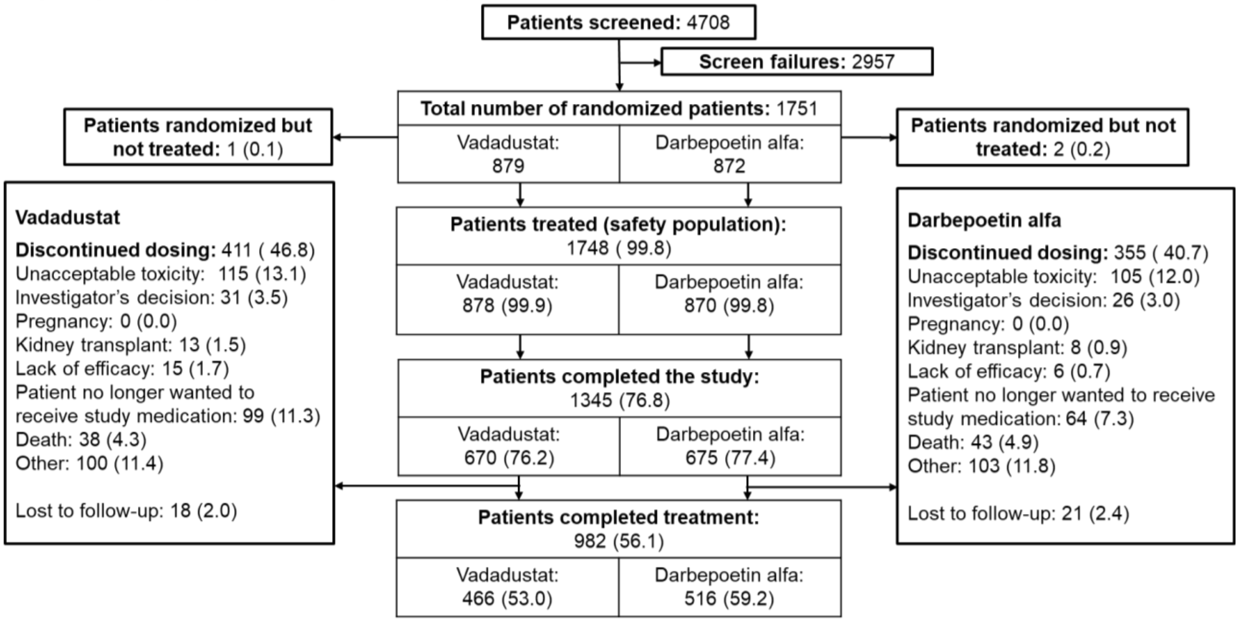

Abbreviations: ESA, erythropoiesis-stimulating agent; NDD-CKD, non–dialysis-dependent chronic kidney disease.

Table S1. Regional Classification of Countries That Enrolled Patients for PRO$_2$TECT ESA-Untreated Trial

**Region**	**US**	**Europe**	**Non-US/non-Europe**
**Countries**	United States	Bulgaria<br>France<br>Hungary<br>Italy<br>Spain<br>United Kingdom	Argentina<br>Australia<br>Brazil<br>Canada<br>Malaysia<br>Mexico<br>New Zealand<br>Russia<br>South Africa<br>South Korea<br>Ukraine

Abbreviation: ESA, erythropoiesis-stimulating agent.

Table S2. Time to First Expanded MACE and All-Cause Mortality in NDD-CKD ESA-Untreated Patients by Region (Safety Population)

	Hazard ratio (vadadustat/darbepoetin alfa) (95% CI)			
**Endpoint**	**Overall**	**US**	**Europe**	**Non-US/non-Europe**
Expanded MACE	1.16 (0.97, 1.38)	1.06 (0.85, 1.33)	0.89 (0.46, 1.69)	1.54 (1.10, 2.17)
All-cause mortality	1.11 (0.90, 1.38)	0.94 (0.70, 1.24)	1.11 (0.50, 2.47)	1.56 (1.10, 2.21)

Abbreviations: CI, confidence interval; ESA, erythropoiesis-stimulating agent; MACE, major adverse cardiovascular event; NDD-CKD, non–dialysis-dependent chronic kidney disease.

Table S3. MACE Rates in NDD-CKD ESA-Untreated Patients by Region With Baseline EGFR ≤10 and >10 mL/min/1.73 m^2

eGFR, mL/min/1.73 m^2		Overall		US		Europe		Non-US/non-Europe	
		Vadadustat N = 878	Darbepoetin alfa N = 870	Vadadustat N = 531	Darbepoetin alfa N = 527	Vadadustat N = 71	Darbepoetin alfa N = 68	Vadadustat N = 276	Darbepoetin alfa N = 275
Overall	No. of patients with MACE, n (%)[a]	214 (24.4)	192 (22.1)	121 (22.8)	119 (22.6)	15 (21.1)	16 (23.5)	78 (28.3)	57 (20.7)
	Hazard ratio (95% CI)	1.16 (0.96, 1.41)		1.05 (0.81, 1.35)		0.84 (0.41, 1.75)		1.57 (1.11, 2.21)	
≤10	No. of patients in eGFR range	158	138	55	67	7	5	96	66
	No. of patients with MACE, n (%)[b]	55 (34.8)	37 (26.8)	16 (29.1)	18 (26.9)	1 (14.3)	1 (20.0)	38 (39.6)	18 (27.3)
	Hazard ratio (95% CI)	1.47 (0.96, 2.27)		1.16 (0.57, 2.39)		N/A		1.79 (1.01, 3.20)	
>10	No. of patients in eGFR range	720	732	476	460	64	63	180	209
	No. of patients with MACE, n (%)[b]	159 (22.1)	155 (21.2)	105 (22.1)	101 (22.0)	14 (21.9)	15 (23.8)	40 (22.2)	39 (18.7)
	Hazard ratio (95% CI)	1.12 (0.89, 1.39)		1.06 (0.80, 1.39)		0.88 (0.42, 1.88)		1.46 (0.93, 2.30)	

Abbreviations: CI, confidence interval; eGFR, estimated glomerular filtration rate; ESA, erythropoiesis-stimulating agent; MACE, major adverse cardiovascular event; N/A, not available; NDD-CKD, non–dialysis-dependent chronic kidney disease.

[a]Percentage of patients experiencing a MACE was calculated by dividing the number of patients with a MACE by the number of patients in the Overall population or the respective region.

[b]Percentage of patients experiencing a MACE was calculated by dividing the number of patients with a MACE by the number of patients in the respective eGFR range.
